# Altered Immunity and Microbial Dysbiosis in Aged Individuals With Long-Term Controlled HIV Infection

**DOI:** 10.3389/fimmu.2019.00463

**Published:** 2019-03-12

**Authors:** Nicholas Rhoades, Norma Mendoza, Allen Jankeel, Suhas Sureshchandra, Alexander D. Alvarez, Brianna Doratt, Omeid Heidari, Rod Hagan, Brandon Brown, Steven Scheibel, Theodore Marbley, Jeff Taylor, Ilhem Messaoudi

**Affiliations:** ^1^Molecular Biology and Biochemistry, University of California Irvine, Irvine, CA, United States; ^2^School of Nursing, John Hopkins University, Baltimore, MD, United States; ^3^Stonewall Medical Center, Borrego Health, Cathedral City, CA, United States; ^4^School of Medicine, University of California, Riverside, Riverside, CA, United States; ^5^HIV+ Aging-Palm Springs, Palm Springs, CA, United States

**Keywords:** HIV, HAART, aging, dysbiosis, inflammation

## Abstract

The introduction of highly active antiretroviral therapy (HAART) resulted in a significant increase in life expectancy for HIV patients. Indeed, in 2015, 45% of the HIV+ individuals in the United States were ≥55 years of age. Despite improvements in diagnosis and treatment of HIV infection, geriatric HIV+ patients suffer from higher incidence of comorbidities compared to age-matched HIV- individuals. Both chronic inflammation and dysbiosis of the gut microbiome are believed to be major contributors to this phenomenon, however carefully controlled studies investigating the impact of long-term (>10 years) controlled HIV (LTC-HIV) infection are lacking. To address this question, we profiled circulating immune cells, immune mediators, and the gut microbiome from elderly (≥55 years old) LTC-HIV+ and HIV- gay men living in the Palm Springs area. LTC-HIV+ individuals had lower frequency of circulating monocytes and CD4+ T-cells, and increased frequency CD8+ T-cells. Moreover, levels of systemic INFγ and several growth factors were increased while levels of IL-2 and several chemokines were reduced. Upon stimulation, immune cells from LTC-HIV+ individuals produced higher levels of pro-inflammatory cytokines. Last but not least, the gut microbiome of LTC-HIV+ individuals was enriched in bacterial taxa typically found in the oral cavity suggestive of loss of compartmentalization, while levels of beneficial butyrate producing taxa were reduced. Additionally, prevalence of *Prevotella* negatively correlated with CD4+ T-cells numbers in LTC-HIV+ individuals. These results indicate that despite long-term adherence and undetectable viral loads, LTC-HIV infection results in significant shifts in immune cell frequencies and gut microbial communities.

## Introduction

Advances in the diagnosis and treatment of HIV infection has transformed what used to be a fatal diagnosis into a long-term chronic disease with individuals living up to 50 years after diagnosis (1, 2). In the United States, 450,000 of those living with HIV are ≥50 years of age, and this number is expected to grow over the coming decade (3). Highly active anti-retroviral therapy (HAART) must be taken continuously to maintain viral loads below detection (4, 5). However, low levels of viral replication can be detected in virtually all patients receiving HAART (6). Moreover, mortality rates among individuals living with long-term controlled HIV infection (LTC-HIV) remains higher than those seen in age-matched HIV- individuals due to HIV-associated non-AIDS (HANA) co-morbidities (7–9). HANA co-morbidities include neurocognitive disorders, cardiovascular disease, cancer, liver cirrhosis, kidney, and lung disease (10–16). The average number of comorbid conditions is higher in older HIV+ patients compared to age-matched HIV- subjects, indicative of accelerated aging (10, 17). The mechanisms underlying this increase in comorbidities and resulting mortality remain unclear. Consequently, very few interventions are available to counteract these adverse outcomes.

Some of these comorbidities could be explained by the high rates of co-infection among LTC-HIV patients, notably with Hepatitis C (18, 19). Moreover, HAART toxicity itself increases the risk of cardiovascular disease and metabolic dysfunction via mitochondrial dysfunction, but this does not fully explain the increase in neurocognitive disorders (20–23). Another potential factor is the increased systemic inflammation reported in LTC- HIV infection (24–27). While the initiation of HAART has been shown to reduce immune activation, the levels remain higher than those observed in HIV- individuals even after 2 years of treatment (28–31). The sources of this systemic inflammation are poorly understood. Some studies have suggested an association between shifts in the gut microbiome and systemic inflammation (32, 33). HIV-induced loss of Th17 CD4+ T cells in the gut is thought to disrupt the microbial community and impair the gut barrier resulting in the translocation of microbial products (34, 35). Indeed, increased levels of sCD14, LPS, IgM, iFABP, and endotoxin-bound IgM have been reported in HIV+ individuals at various stages of infection (36, 37). Some studies have reported an increase in the abundance of *Prevotella* in untreated, and HAART treated HIV+ individuals while others reported no changes (33, 38). Furthermore, disruption in tryptophan metabolism, reduction in short chain fatty acid (SCFA) production, and an increase in trimethylamine-n-oxide (TMAO) production have all been linked to increased HANA co-morbidities (39–41).

Our understanding of the role of circulating immune cells in the development and exacerbation of systemic inflammation also remains incomplete. While many have reported some recovery of CD4+ T cells upon initiation of HAART, this finding has only has been explored through the first 5 years of treatment (42–44). An increase in terminally differentiated T cells has also been listed as a potential source of aberrant immune activation (45). CD4+ T cells are not the only immune cells affected by HIV infection. Monocytes in particular are activated directly during initial infection by circulating viral proteins such as gp120 and Nef (45) and indirectly at later stages due to an increase in bacterial products such as LPS in circulation (46–48). Activated monocytes can release multiple immune mediators such as TNFα, IL-1β, IL-6 that can contribute to systemic inflammation (49). Natural Killer (NK) cells also play an important role in controlling HIV through the production of INFγ which suppresses infection, but can also contribute to excessive inflammation (50). HAART has been shown to restore NK populations over the first couple of years of treatment, but the durability of this recovery is not well-understood (51).

The lack of clear understanding of the impact of LTC-HIV infection on immune status, systemic inflammation, and microbiome communities is likely due to the paucity of well-controlled studies. Researchers often compare HIV+ and HIV- groups of differing socioeconomic status, sexual orientation, and geographic location. These factors can affect immunological status and the gut microbiome. To address this limitation, we obtained blood and rectal swabs from older (≥55 years of age) LTC-HIV+ gay men (>10 years post-infection) and HIV- participants residing in the Palm Springs area. Our analysis shows that despite adherence to HAART treatment, HIV status is associated with a reduction in circulating CD4+ T-cells and monocytes, an increase in CD8+ T-cells, and greater activation/differentiation of NK cells. We also detected signs of heightened systemic inflammation and induced inflammatory response to polyclonal stimulation. Surprisingly, we detected a limited number of shifts in the gut microbial community with an increase in Fusobacteria, Bifidobacteriales, and Lactobacillales in LTC-HIV+ men. *Prevotella* was negatively correlated with CD4+ T-cell count in LTC-HIV+ but not HIV- individuals. Together these data suggest that significant differences in immunological and microbial communities persist in LTC-HIV infection.

## Methods

### Study Population and Sample Collection

The Institutional Review Board of the University of California, Riverside reviewed and approved this study, and the University of California, Irvine obtained a reliance registry.

A total of 105 men ≥55 years of age (HIV- *n* = 47, LTC-HIV+ *n* = 58) were recruited from Palm Springs, CA. All LTC-HIV+ individuals have been on HAART therapy for >10 years with undetectable viral loads. Median CD4+ cell counts in the LTC-HIV cohort were 689 cells/mm^3^ with only two individuals having CD4+ T cell count <200 cells/mm^3^ while CD4+ cell counts in the HIV- cohort were 1198 cells/mm^3^. Each participant submitted a blood sample, a rectal swab, and completed a paper demographics/co-morbidities survey (Table 1). Complete blood cell counts (CBC) were determined using a hematology analyzer (Beckman Coulter). Peripheral blood mononuclear cells (PBMC) and plasma were obtained by standard density gradient centrifugation over the blood separation polymer Ficoll (GE Healthcare Life Sciences, Pittsburg, PA, United States). PBMC were frozen in 10% DMSO/FBS and stored in liquid nitrogen while plasma was stored at 80°C until analysis.

**Table 1:** Demographics and co-morbidities.

	**Total *n* [*S.D*.](%)**	**LTC-HIV + *n* [*S.D*.](%)**	**HIV – *n* [*S.D*.](%)**	***p***
**DEMOGRAPHICS**				
Age	64 [6.8]	65.7 [7.3]	62.7 [6.03]	0.02^*^
Race				0.82
Non-hispanic white	90 (84.6)	49 (84.5)	41 (87.2)	
Other	15 (15.5)	9 (15.5)	6 (12.8)	
Education				0.299
High school	8 (8.3)	7 (12.5)	1 (2.4)	
Some college	32 (33.3)	21 (37.5)	11 (26.8)	
Four-year university	25 (24.0)	13 (23.2)	12 (29.3)	
Graduate school	32 (33.3)	15 (26.8)	17 (41.4)	
Relationship status				0.10
Single	43 (41.7)	29 (49.2)	14 (31.8)	
In a relationship	18 (17.5)	10 (16.9)	8 (18.2)	
Engaged	2 (1.9)	1 (1.7)	1 (2.3)	
Married	34 (33.0)	14 (23.7)	20 (45.5)	
Widowed	6 (5.8)	5 (8.5)	1 (2.3)	
On disability	28 (28.28)	25 (43.86)	3 (7.14)	0.001^***^
**COMORBIDITIES**				
Depression	27 (25.96)	21 (35.59)	6 (13.33)	0.013^*^
Arthritis	30 (28.85)	17 (28.81)	13 (28.89)	1.000
Hepatitis	8 (7.69)	8 (13.56)	0 (0)	0.009^***^
Neuropathy	33 (31.73)	24 (40.68)	9 (20)	0.033^*^
Hypertension	38 (36.54)	18 (30.51)	20 (44.44)	0.156
Dermatitis	12 (11.54)	10 (16.95)	2 (4.44)	0.064
Herpes	13 (12.50)	8 (13.56)	5 (11.11)	0.773
Vision loss	17 (16.35)	9 (15.25)	8 (17.78)	0.792
Diabetes	13 (12.5)	8 (13.56)	5 (11.11)	0.773
Hearing loss	25 (24.04)	11 (18.64)	14 (31.11)	0.168
Respiratory problems	6 (5.78)	3 (5.08)	3 (6.67)	1.000
Heart condition	11 (10.58)	4 (6.78)	7 (15.56)	0.201
Broken bones	1 (0.96)	0 (0)	1 (2.22)	0.433

Chi-squared or Fisher's exact tests were conducted to determine significant differences between the two

* *p < 0.05*,

*** *p < 0.001*.

### Flow Cytometry

1–2 × 10^6^ PBMC were stained using antibodies against: CD4, CD8b, CD95, CD28, CCR7, CD20, CD27, IgD, PD-1, KLRG1, and Ki-67 to delineate naïve and memory T and B cell populations, as well as measure activation and proliferation (Supplementary Figure 1). A second tube of 1–2 × 10^6^ PBMC was stained using antibodies against: CD3, CD20, HLA-DR, CD14, CD11c, CD123, CD56, CD16, granzymeB, and CD57 to delineate monocytes, myeloid dendritic cells (mDC), plasmacytoid dendritic cells (pDC), and natural killer (NK) cell subsets, respectively (Supplementary Figure 2). All flow cytometry samples were acquired with Attune NxT (Life Technologies, Carlsbad, CA, United States) and analyzed using FlowJo (TreeStar, Ashland, OR, United States).

### PBMC Stimulation

5 × 10^5^ PBMC were incubated for 16 h in the absence (unstimulated) or presence (stimulated) of 5 ng/ml PMA and 1 μg/ml Ionomycin at 37°C in a humidified incubator (5% CO_2_). At the end of the incubation, cells were spun down for 5 min at 2,000 rpm. Supernatants were collected to measure production of immune mediators and cell pellets were used for RNA extraction.

### Analysis of Immune Mediator Production

Plasma levels of immune mediators were assessed using a human 45-plex assay (Thermo Fisher Scientific, Waltham, MA, United States). Immune mediators produced by PBMC in response to PMA/ionomycin stimulation were measured using Magnetic 33-plex assay (R&D Systems, Minneapolis, MN, United States). All samples were analyzed using a MAGPIX instrument (Luminex, Austin, TX, United States).

### RNAseq Library Prep

Total RNA was isolated from PBMC pellets obtained from control and stimulated wells using the Qiagen mRNAeasy kit (Qiagen, Valencia, CA, United States). RNA concentration and integrity was verified using Agilent 2,100 Bioanalyzer. Total RNA was depleted of ribosomal fraction using Ribo-Gone rRNA removal kit. Libraries were then constructed using SMARTer Stranded RNA-Seq Kit (Clontech, Mountain View, CA, United States). Following assessment of size, quality, and concentrations, libraries were multiplexed and subject to sequencing (75 bp single end) on the NextSeq platform (Illumina, San Diego, CA, United States).

### RNAseq Data Analysis

Quality control of raw reads was performed retaining bases with quality scores of 20 and reads at least 50 base pair long. Reads were aligned to human genome (hg38) using splice aware aligner TopHat as previously described (52) using annotations available from Ensembl (GRCh38.85) database. Quantification and statistical validation of differentially expressed genes (DEGs) was performed using edgeR (https://bioconductor.org/packages/release/bioc/html/edgeR.html), with candidate genes defined by at least two-fold change in expression with multiple hypothesis corrected (Benjamini-Hochberg procedure) FDR < 0.05. Functional enrichment of DEGs was performed using open source functional enrichment tool Metascape (53) and disease associations analyzed using MetaCore™. Heatmaps were generated in R. Gene expression data have been deposited in NCBI's Sequence Read Archive BioProject (PRJNA518909).

### 16S rRNA Gene Library Construction and Sequencing

Total DNA was extracted from rectal swabs using the PowerSoil DNA Isolation Kit (MO BIO Laboratories, Carlsbad, CA, USA), and a 30-s bead-beating step using a Mini-Beadbeater-16 (BioSpec Products, Bartlesville, OK, USA). This genomic DNA was used as the template to amplify the hypervariable V4 region of the 16S rRNA gene using PCR primers (515F/806R with the reverse primers including a 12-bp barcode) and reactions containing: 50 mM Tris (pH 8.3), 500 μg/ml bovine serum albumin (BSA), 2.5 mM MgCl2, 250 μM of each deoxynucleotide triphosphate (dNTP), 400 nM of each primer, 5 μl of DNA template, and 0.25 units of JumpStart Taq DNA polymerase (Sigma-Aldrich, St Louis, MO, USA). Thermal cycling parameters were 94°C for 5 min; 35 cycles of 94°C for 20 s, 50°C for 20 s, 72°C for 30 s, followed by 72°C for 5 min. PCR products were purified using a MinElute 96 UF PCR Purification Kit (Qiagen, Valencia, CA, USA). Libraries were sequenced (1 × 300 bases) using an Illumina MiSeq.

### 16S rRNA Gene Sequence Processing

Raw FASTQ sequence files of 16S rRNA genes were processed with the UPARSE pipeline (54). We used a custom Python script to demultiplex and prepare sequence files for clustering. Sequences were then filtered at a maxee value of 1.0 (allowing only one nucleotide per sequence to be incorrect) to remove low-quality sequences. The remaining sequences were dereplicated and singletons were removed. Operational taxonomic units (OTUs) were assigned with UPARSE at a 97% sequence identity threshold. Taxonomy was assigned to sequences for each OTU using the Ribosomal Database Project (RDP) classifier with a confidence threshold of 0.5 against the Greengenes 13_8 database using Quantitative Insights Into Microbial Ecology (QIIME) version 1.9.1 (55). To eliminate any bias due to sequencing depth, all samples were rarefied to 13,000 sequences per sample before calculating diversity metrics. 16S amplicon sequencing data have been deposited in NCBI's Sequence Read Archive BioProject (PRJNA518910).

## Results

### Long-Term HIV Infection Is Associated With Significant Shifts in Circulating Immune Cell Populations.

It has been well-established that HIV infection is associated with alterations of lymphocyte subsets both in circulation and lymphoid tissues (56). This can be partially remediated using HAART (57), but the long-term outcomes of HAART (>10 years) on immune cell frequencies has not been addressed. To this end, we compared frequencies of circulating lymphocyte and myeloid populations within peripheral blood mononuclear cells (PBMC) between HIV- and LTC-HIV+ older men (≥55 years of age). As previously reported, our CBC data showed a reduced number of total lymphocytes in the blood with LTC-HIV infection (Supplementary Figure 3C) with no additional changes in total leukocytes, granulocytes or monocyte numbers (Supplementary Figures 3A,B,D). Since CBC data is based on cell size and granularity rather than specific cell markers, we next investigated the impact of LTC-HIV infection on circulating immune population via flow cytometry.

**Figure 1:**
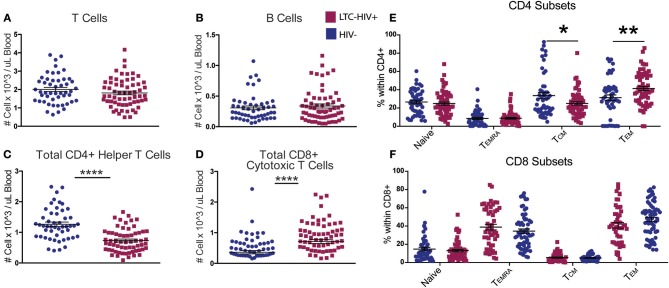
LTC-HIV results in alterations in T but not B cell subsets. **(A)** Numbers of circulating CD3+ T cells **(B)** CD20+ B cells **(C)** CD3+CD4+ helper T cells, and **(D)** CD3+CD8+ cytotoxic T cells. **(E,F)** Relative frequencies of CD4+ **(E)** and CD8+ **(F)** T cell subsets **(E)**. Each point represents a study subject. Horizontal bars and whiskers indicate the mean ± SEM. Significance was determined using an unpaired *t*-test. ^*^p < 0.05, ^**^p < 0.01, ^****^p < 0.0001.

Total T and B cell numbers (CD3+ and CD19+, respectively) did not differ significantly between the two groups (Figures 1A,B). However, a significant reduction in the number of CD4+ helper T cells was detected in LTC-HIV+ individuals (Figure 1C). Conversely, the number of circulating CD8+ cytotoxic T cells was increased in the LTC-HIV+ group (Figure 1D). We next determined the effect of LTC-HIV infection on the relative frequencies of naïve, transitional effector memory (TEMRA), central memory (TCM), and effector memory (TEM) (Supplementary Figure 1). This analysis showed that the frequency of CD4+TCM cells was significantly reduced, while the frequency of CD4+TEM cells was significantly increased (Figure 1E). No changes in frequency of naïve and memory CD8+ T cell subsets were detected (Figure 1F). Moreover, no changes in the frequency of naïve and memory B cell subsets were noted (Supplementary Figures 3E–G). In terms of homeostatic proliferation, we detected an increase in the TEM CD8+ cells subset but none of the other subsets (Supplementary Figures 4A–D). No differences in homeostatic proliferation of CD4+ subsets were detected (Supplementary Figures 4E–H). Interestingly, we found no differences in T cell activation measured via KLRG1 expression levels, or exhaustion measured via PD-1 expression levels, in either CD4+ (Supplementary Figures 5A,B) or CD8+ (Supplementary Figures 5C,D) T cell subsets.

**Figure 2:**
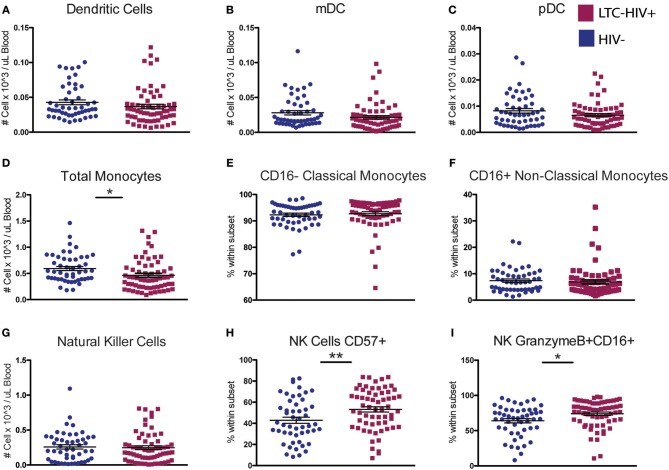
A reduction in circulating monocytes and increase in NK cell activation seen with LTC-HIV infection. **(A)** Number of total circulating dendritic cells (DC) **(B)** myeloid DCs, and **(C)** plasmacytoid (DC). **(D)** Number of total circulating CD14+ monocytes and **(E)** relative abundance of CD16- Classical monocytes, and **(F)** CD16+ non-classical monocytes. **(G)** Number of total circulating CD56+ natural killer (NK) cells and **(H)** relative frequency of CD57+ NK cells and **(I)** granzymeB+CD16+ cytotoxic NK cells. Horizontal bar and whisker indicate the mean ± SEM. Significance was determined using an unpaired *t*-test. ^*^p < 0.05, ^**^p < 0.01.

We next characterized the frequency of circulating innate immune cells. No significant differences were seen in the abundance of total dendritic cells (DC) or in the relative frequencies of myeloid DC (mDC) and plasmacytoid DC (pDC) with LTC-HIV infection (Figures 2A–C). In contrast, the number of circulating monocytes was reduced in LTC-HIV+ individuals (Figure 2D), with no differences observed within monocyte subsets (Figures 2E,F). Lastly, no differences were detected in natural killer (NK) cell frequencies (Figure 2G). However, the relative abundances of activated CD57+ and cytotoxic CD16+GrzmB+ NK cells were higher in LTC-HIV+ group (Figures 2H,I).

### Long-Term HIV Infection Leads to a Heightened Pro-Inflammatory Response at Baseline and After Stimulation

**Figure 3:**
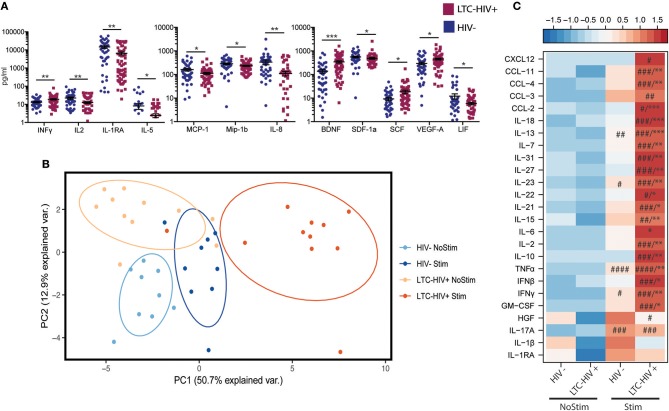
Change in circulating and production of immune mediators following stimulation. **(A)** Serum level of immune mediators measured using a multiplexed ELISA assay. Horizontal bar and whiskers indicate the mean ± SEM. Significance was determined using an unpaired *t*-test. ^*^p < 0.05, ^**^p < 0.01, ^***^p < 0.001 **(B)** Principal component analysis of immune mediators released by PBMC's from HIV- and LTC-HIV+ individuals in the absence and presence of PMA/ionomycin. **(C)** Heat map of differentially abundant immune mediators produced by PBMCs upon stimulation (all immune mediators statistically significant via 1-way ANOVA, Significant Tukeys *post-hoc* comparison of stimulated samples vs. corresponding baseline samples) #*p* < 0.05, ##*p* < 0.01, ###*p* < 0.001, ####*p* < 0.0001. Unpaired *T*-tests of absolute change followed by Welch's correction [Stimulation—baseline] in immune mediator production ^*^p < 0.05, ^**^p < 0.01, ^***^p < 0.001.

To explore the impact of LTC-HIV on systemic inflammatory markers, we measured plasma levels of cytokines, chemokines, and growth factors using Luminex technology. This analysis revealed significantly higher levels of the pro-inflammatory cytokine INFγ as well as growth factors BDNF, SCF, and VEGF (Figure 3A). On the other hand, levels of anti-inflammatory cytokines IL-1RA, T cell cytokine IL-2, eosinophil maturation factor IL-5, inhibitory cytokine LIF, growth factor SDF1a, as well as chemo-attractants MCP-1, MIP-1b, and IL-8 were reduced in LTC-HIV+ individuals (Figure 3A).

To further explore the effect of LTC-HIV infection on immune mediator production, we stimulated a subset of PBMC samples (HIV- *n* = 9, LTC-HIV *n* = 11) with PMA/ionomycin for 16 h and measured the production of cytokines, chemokines, and growth factors via Luminex technology. Samples from LTC-HIV patients generated a more pronounced and variable response to stimulation (Figure 3B). After correcting for baseline spontaneous production, 21 of 33 measured analytes were produced in higher amounts in response to stimulation by samples from the LTC-HIV+ group compared to the HIV- group. These included the pro-inflammatory cytokines IL-6, TNFα, INFγ as well as anti-inflammatory cytokine IL-10 (Figure 3C).

**Figure 4:**
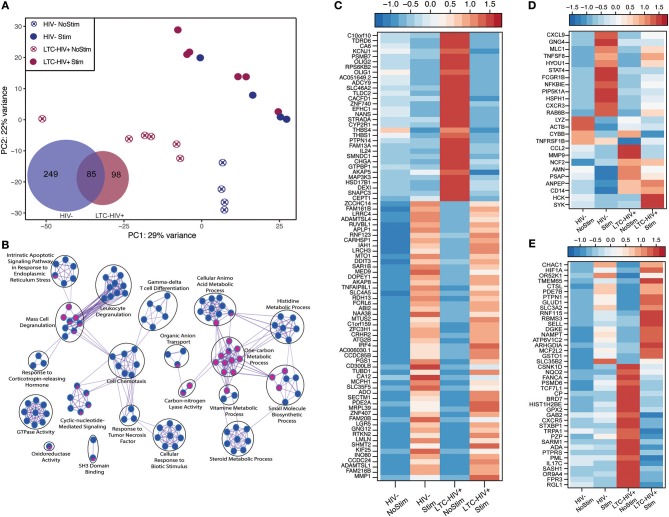
LTC-HIV infection alters PBMC transcriptional response to PMA/Ionomycin. **(A)** Principal component analysis of unstimulated and PMA/ionomycin stimulated PBMCs from HIV- and LTC-HIV and Venn diagram of stimulation DEGs showing unique and common protein coding genes. **(B)** Network visualization using Cytoscape of functional enrichment output of DEGs detected in PBMC from LTC-HIV+ and HIV- subjects following PMA stimulation obtained using Metascape. The size of node represents the number of DEGs associated with each gene ontology (GO) term and the pie chart filling represents relative proportion of each group's DEGs that enriched to that GO term. Heat maps of select DEGs for Common **(C)**, HIV- **(D)**, and LTC-HIV+ **(E)**.

We next interrogated whether the increased response of LTC-HIV PBMCs to PMA/ionomycin was mirrored by an increased transcriptional response using RNA sequencing (RNA-Seq). Principal component analysis (PCA) shows that the samples clustered based on group and treatment (Figure 4A). At baseline, we identified only 11 protein coding differentially expressed genes (DEGs) all of which were down-regulated in LTC-HIV samples (*TNFRSF1B, IL7R, ARHGDIB, RPS27, PPBP, CTSL, HIST1H2BJ, TNKS2, TTC37, SEC24B*, and *EEF1A1)*. Following PMA/ionomycin stimulation, a larger number of DEGs were detected in the HIV- group (334) compared to the LTC-HIV+ group (183), with 85 DEGs detected in both groups (Figure 4A). To better understand the biological significance of these changes, we carried out an initial functional enrichment using Metascape (53). DEGs detected in the samples from the HIV- group enriched to gene ontology (GO) terms associated with host defense including “cell chemotaxis,” “response to TNF,” and “leukocyte degranulation” (Figure 4B). On the other hand, DEGs detected in the LTC-HIV+ group enriched to GO terms associated with metabolic processes (Figure 4B). DEGs detected in both groups enriched to GO terms associated with signaling (Figure 4B).

Additional functional enrichment using Metacore™ showed that the 85 common DEGs enriched to GO terms such as “response to LPS” and “response to molecule of bacterial origin” characteristic of a response to antigenic stimulation (Table 2). Several of 32 genes that were downregulated upon stimulation in both groups were important for cell adhesion, and proliferation such as: *IL-24, ZNF740, MAP3K3, THBS1*, and *THBS4* (Figure 4C). Some of the 53 genes that were upregulated in both groups include genes important for myeloid activation and interferon regulation such as: *CD300LB, TNFAIP8L1, MMP1, SLC35F5*, and *IRF4* (Figure 4C).

**Table 2:** Functional enrichment of DEGs detected in response to PMA stimulation.

	**No of genes**	**FDR *p*-value**
**COMMON DEGs**		
Regulation of purine nucleotide metabolic process	12	1.517E-05
Response to lipopolysaccharide	10	1.565E-02
Response to molecule of bacterial origin	10	1.888E-02
Regulation of phosphorus metabolic process	21	2.026E-02
**DEGs DETECTED IN LTC-HIV SAMPLES ONLY**		
Regulation of signaling	46	1.711E-03
Neutrophil homeostasis	23	2.506E-03
Regulation of communication	44	2.506E-03
Regulation of blood circulation	11	2.574E-03
**DEGs DETECTED IN HIV- SAMPLES ONLY**		
Intracellular signal transduction	63	1.190E-06
Cell chemotaxis	19	1.335E-06
Response to cytokine	52	1.740E-06
Inflammatory response	32	2.492E-06

In line with the output of Metascape, functional enrichment using Metacore™ showed that DEGs that were only detected in PBMCs from HIV- individuals enriched to GO terms such as “inflammatory response,” and “response to cytokines” (Table 2). Some of the notable DEGs that were only significantly up-regulated in PBMCs from HIV- individuals included: *CXCL9, TNFSF8, NFKBIE, CXCR3*, and *STAT4* (Figure 4D). Downregulated DEGs that enriched to the same GO terms included: *LYZ, CCL2*, and *CD14* (Figure 4D). Similar to the Metascape output, DEGs that were only detected in PBMC from the LTC-HIV+ group enriched to GO terms associated with signaling (Table 2). Genes that were uniquely up-regulated in PBMCs from LTC-HIV+ individuals included: *HIF1A, DHE3*, and solute carrier gene *SLC3A2* which are unique to CD8+ T cells as well as *CXCR5, IL17C*, and *TRPA1* (Figure 4E).

To elucidate the source of the DEGs detected, we used the Immunological Genome Project Consortium database (ImmGen), which delineates gene expression patterns across different leukocyte subsets (58). This analysis revealed that DEGs detected only in LTC-HIV+ samples are predicted to be expressed by DCs, monocytes, macrophages, and to a lesser extent T cells and B cells (Supplementary Figure 6A). DEGs detected only in the HIV- samples were mostly expressed by monocytes/macrophages and DCs (Supplementary Figure 6B), while common DEGs exhibited broad distribution (Supplementary Figure 6C). To gain a better understanding of alterations in immune cells frequencies following stimulation, we employed Immquant, which uses the digital cell quantification algorithm (59) to predict changes in immune cell subsets based on total transcriptional profiles using the IRIS database (60). This analysis predicted that the transcriptional changes observed were associated with a significant increase of stimulated Th1 CD4+ T cells in both LTC-HIV+ and HIV- individuals upon PMA stimulation (Supplementary Figure 6D). Additionally, transcriptional changes in LTC-HIV+ individuals were associated with an increase in stimulated Th2 CD4+ T cells and LPS stimulated DCs upon PMA stimulation (Supplementary Figure 6D).

### Long-Term HIV Infection Leads to Dysbiosis

Several studies have suggested that HIV infection results in increased gut permeability and translocation of microbial products into circulation (46, 47, 61, 62). To determine if those changes were evident in an older population with LTC-HIV infection, we evaluated several markers of barrier function. Analysis of circulating levels of sCD14, fatty acid binding protein (iFABP), IgM-bound endotoxin and Limulus amebocyte lysate (LAL) did not reveal significant differences between the two group (Supplementary Figures 7A–D).

**Figure 5:**
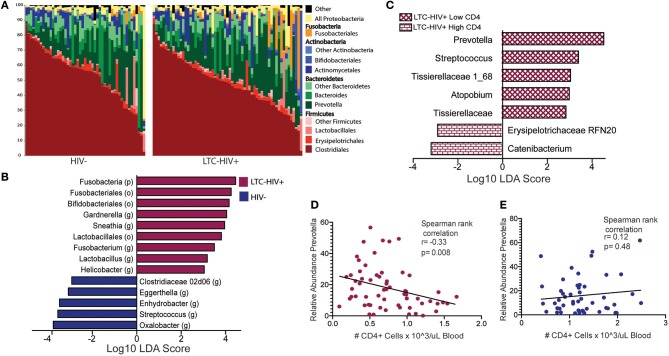
Taxonomic differences in the gut microbiome of LTC-HIV **(A)** Stack bar plot illustrating the abundance of bacterial orders and phyla in HIV- and LTC-HIV+ individuals. **(B)** Differentially abundant bacterial taxa between LTC-HIV+/HIV- determined using LEfSE (Log_10_ LDA score >2) at the Phyla, Order and Genus level. **(C)** Differentially abundant genera between LTC-HIV+ High CD4+ and LTC-HIV+ Low CD4+ individuals (Log_10_ LDA score > 2). **(D)** Spearman rank correlation between CD4+ T cell abundance and relative abundance of *Prevotella* in LTC-HIV+ individuals. **(E)** Spearman rank correlation between CD4+ T cell abundance and relative abundance of *Prevotella* in HIV- individuals.

Next, we investigated whether LTC-HIV infection is associated with alterations in the gut microbiome using 16S amplicon sequencing. The overall taxonomic landscape of the gut microbiome was not significantly different as measured by alpha- and beta-diversity (Figure 5A, Supplementary Figures 7E–G). Specifically, principal coordinate analysis (PCoA) of unweighted UniFrac distance showed significant overlap in the overall gut microbiome communities of HIV- and LTC-HIV+ individuals (Supplementary Figure 7E). Both groups also had similar numbers of observed species and Pielou evenness (Supplementary Figures 7F,G).

At the phyla level, microbiomes from both groups were dominated by Firmicutes and Bacteroidetes with smaller contributions from Actinobacteria and Proteobacteria (Figure 5A). Fusobacteria was the fourth most abundant phyla across all samples and significantly enriched in LTC-HIV+ individuals (Figure 5B). Clostridiales and Bacteroidales were the most abundant bacterial orders and *Prevotella* was the most abundant genus across both groups (Figure 5A). Ten genera were differentially abundant between LTC-HIV+ and HIV- individuals. The gut microbiomes of LTC-HIV+ individuals were enriched in; *Garnerella, Snethia, Fusobacterium, Lactobacillus*, and *Helicobater* (Figure 5B). While the gut microbiomes of HIV- individuals had a significantly higher relative abundance of; *Oxalobacter, Streptococcus, Enhydrobacter, Eggerthella*, and *Clostridiaceae* 02d06 (Figure 5B).

To further explore the relationship between the microbial communities of the gut microbiome and LTC-HIV, we stratified our LTC-HIV+ individuals into quartiles based on the CD4+ T cell numbers and compared the microbial communities of individuals in the highest and lowest quartile. This analysis revealed seven differentially abundant genera between the highest and lowest CD4+ T cell quartiles (Figure 5C). Of note, *Prevotella* was significantly more abundant in individuals with a low CD4+ individuals (Figure 5C) and negatively correlated with CD4+ T cell count across all LTC-HIV+ individuals (Figure 5D), a pattern not seen in HIV- individuals (Figure 5E).

## Discussion

The advent and rapid adoption of HAART treatment in the U.S. has turned HIV infection into a chronic disease. Indeed, the average life expectancy of individuals diagnosed with HIV at age 20 has risen from 32 in the late 1980's to 71 in 2018 (63). Consequently, the majority of HIV+ individuals in the U.S. are expected to be older than 50 years of age by 2020 (64). However, HAART treatment is associated with significant adverse outcomes, and individuals who have received long-term HAART are experiencing a higher incidence of several morbidities compared to age-matched HIV- individuals (17). These HANA co-morbidities include cardiovascular disease, neurocognitive disorders, osteoporosis, cancer, liver cirrhosis, kidney, and lung disease (10–16). The mechanisms underlying this increased susceptibility are poorly understood due to the lack of carefully controlled studies on older HIV+ individuals who have been on HAART for >5 years. In this study, we addressed this question using samples collected from a large cohort of LTC-HIV+ gay men who have received HAART treatment for at least 10 years. Importantly, the control population was matched with the LTC-HIV+ cohort for age, sexual preference, location, and socioeconomic status. This experimental set up allowed us to determine the effects of LTC-HIV infection on immunological and microbial shifts that can be subsequently targeted for intervention to reduce HANA co-morbidities.

HAART treatment within the first 3 years results in a rebound in CD4 T cell count that is strongly linked to the CD4 count at the time of treatment initiation (42–44, 57). However, our data showed a significant reduction in total circulating CD4+ T cells. Thirty-six of the fifty-seven participants in this study were initially infected with HIV in the 1980's well before the introduction of HAART. Consequently, 28 of these individuals did not have access to therapy for ~10 years prior to starting this treatment and likely began with very low CD4+ counts. Unfortunately we did not have access to the nadir CD4+ counts for our study population, which were shown to be predictive of CD4+ levels during HAART treatment (42, 65, 66). Levels of IL-2, a cytokine critical for T cell survival were also reduced and could partially explain the reduced CD4+ T cell numbers we observed in LTC-HIV+ individuals (67). Both aging and long-term HIV infection have been associated with an accumulation of terminally differentiated T cells (68, 69). In line with these earlier studies, we found a greater shift toward terminally differentially CD4+ T cells in this group indicative of accelerated differentiation of memory T cells.

CD8+ T cells play an important role in the control of HIV infection and their numbers become elevated rapidly upon HIV infection and persist after initiation of HAART (70, 71). Although the numbers of CD8+ T cells were significantly higher in LTC-HIV+ individuals, no differences were observed in the relative abundance of naïve and memory subsets. We did however detect a significant increase in homeostatic proliferation within the CD8+ T cell EM subset, suggestive of accelerated conversion to memory T cells. Additionally, a large percentage of circulating NK cells showed higher activation with LTC-HIV infection. Collectively, this increased differentiation/proliferation of circulating lymphocytes could contribute to heightened systemic inflammation in LTC-HIV infection. Analysis of circulating immune mediators showed increased serum levels of the antiviral cytokine INFγ. This is in line with previous studies that reported increased levels of INFγ, but not IL-6 and TNFα in HAART treated HIV patients (49). The source of INFγ could be the activated NK cells, CD8+ T cells, or TEM CD4+ T cells, but testing this hypothesis will require additional studies such as single cell RNA sequencing.

The effect of LTC-HIV infection on the innate arm of the immune system is not well-understood. Acute HIV infection is associated with a dramatic increase in inflammatory (non-classical CD16+) monocytes (28). This population has been shown to remain elevated throughout the first year of HAART treatment (72). In contrast, we observed a reduction in total circulating monocytes, and no differences in the relative proportions of CD16+ and CD16- monocytes. Monocytes are susceptible to HIV infection and can serve as a reservoir for latent HIV (48). The reduction in circulating monocyte numbers could potentially be due to low levels of viral reactivation (73), or alternatively mediated by lower levels circulating chemokines MCP-1, MIP-1b, and IL-8. Further analysis of the functional capacity (e.g., phagocytosis and wound healing) of monocytes is needed to assess their contribution to the development of HANA co-morbidities.

We also observed a substantial increase in circulating BDNF, and VEGF-A in the serum of LTC-HIV. Both of these factors have been previously linked to HIV associated neurological disorders including depression (74, 75). HIV protein gp120 has been shown to both increase the production and negatively alter neuronal processing of BDNF (76, 77). It is possible that the buildup of circulating BDNF is a compensatory mechanism due to decreased bioavailability of BDNF in the brain. The continuation of this process despite low viral load seen in LTC-HIV could explain the higher incidence of depression and neuropathy that we and others have observed (78–80). We also detected a reduction in the anti-inflammatory cytokine IL-1RA, which could indicate a higher level of systemic inflammation. These changes are consistent with, but are not as pronounced as what has been reported in HIV+ patients on HAART for a shorter duration (81) or during acute infection prior to the initiation of HAART treatment (82).

Upon polyclonal activation of PBMC we observed a significantly heightened inflammatory response by PBMCs from LTC-HIV individuals. While our study design does not allow us to decisively identify the source of these immune mediators, these data clearly indicate functional abnormalities in circulating immune populations. HIV infection has been shown to alter the epigenetic landscape of both T-cells and monocytes during acute and HAART controlled infections (83–85). Future studies should investigate the link between these epigenetic changes and aberrant inflammatory and anti-microbial responses. Interestingly, our transcriptional analysis showed a smaller number of DEGs in PBMC from LTC-HIV+ subjects despite a greater production of immune mediators. Moreover, while DEGs detected in PBMC from HIV- subjects enriched to GO terms associated with leukocyte activation, those detected in LTC-HIV+ samples enriched to GO terms associated with signaling. One possible explanation is that the response to stimulation peaked earlier in the LTC-HIV+ samples. While the luminex data captures accumulation of immune mediators, RNA-Seq only provides a snap shot of the specific time point selected. Future studies can address this question through a kinetic analysis.

Dysbiosis in the gut microbiome during HIV infection has been implicated as a source of aberrant immune activation and inflammation in acute infection and chronic HIV (32, 33, 86). The host immune system tightly regulates the large reservoir of microbes in the human gut; and loss of key immune cells due to HIV infection (87) can result in changes in composition of microbial communities and their compartmentalization. However, data on HIV-associated shifts in the gut microbiome have been inconsistent likely due to the lack of studies that appropriately controlled for race/ethnicity, diet, sanitary conditions, sexual habits, and socioeconomic status. In contrast, our samples were collected from a cohort of gay, mostly Caucasian, men living in a single geographic location of similar age and socioeconomic status. Analysis of the gut microbial community revealed largely comparable communities with only 10 differentially abundant genera. Fusobacteria, Lactobacillus, and multiple Bifidobacteriales genera were all enriched in LTC-HIV+ individuals. Since these genera primarily colonize the mouth, their presence in the gut microbial communities could signal de-compartmentalization has previously been shown to result in local immune activation (88). Additionally, identical strains of *Fusobacterium* have been found in the oral cavities, and tumors of patients with colorectal cancers (89).

Previous studies reported an increased abundance of the bacterial genus *Prevotella* in LTC-HIV+ individuals; however, many of these studies did not control for sexual orientation, and geographic location, which can influence abundance of *Prevotella* (90, 91). Although we did not find a difference in the abundance of *Prevotella* based on HIV status, we observed a negative correlation between CD4+ T cell counts and *Prevotella* abundance. *Prevotella* has previously been linked to high circulating levels of trimethylamine-N-oxide (TMAO) a compound strongly associated with cardiovascular disease (92). Future metabolomics, and transcriptomic studies will be needed to determine if this higher abundance of *Prevotella* leads to more inflammatory microbial products and if this activity varies in LTC-HIV vs. age matched controls. Abundance of other bacterial genera such as *Streptococcus* and *Atopbium* was also negatively correlated with CD4+ T cell counts and are both commonly found in the oral cavity (93). On the other hand, abundance of the short chain fatty acid (SCFA)-producing Clostridia *Catenibacterium* was higher in LTC-HIV+ individuals in the highest CD4+ T cell quartile. SCFAs produced by gut microbes play an important role in maintaining gut homeostasis and local immune function (94–96). While the abundances of these taxa were too sparse to obtain meaningful correlations with CD4+ T cell counts, this could indicate a greater capacity for beneficial SCFA production in LTC-HIV+ patients with a higher CD4+ count.

In summary, this study provides novel insight into the impact of LTC-HIV infection on systemic immunity and microbial gut communities in older patient populations (≥55 years). A little over half of the subjects in our study were initially infected prior to the advent of HAART. This delay may have interfered with the restorative effect of HAART as shown by a lower abundance of CD4+ T cells and monocytes when compared to age-matched HIV- individuals; immune dysregulation, as evidenced by differences in circulating immune mediators and a more pronounced response to stimulation in LTC-HIV; and taxonomic shifts in the gut microbiome indicative of loss of compartmentalization. Taken together these data provide a more complete picture of the immune and microbial landscape of LTC-HIV infection in an aging population, thereby providing potential avenues for intervention and prevention of HANA co-morbidities.

## Data Availability

The datasets generated for this study can be found in SRA, PRJNA518909, PRJNA518910.

## Author Contributions

IM, JT, TM, StS, RH, and BB contributed conception and design of the study. NM, AJ, SuS, AA, BD, and NR generated and analyzed data. OH and NR performed the statistical analysis. NR and IM wrote the first draft of the manuscript. All authors contributed to manuscript revision, read, and approved the submitted version.

### Conflict of Interest Statement

The authors declare that the research was conducted in the absence of any commercial or financial relationships that could be construed as a potential conflict of interest.
