# Supplementary material for: Altered Immunity and Microbial Dysbiosis in Aged Individuals With Long-Term Controlled HIV Infection
Source: Front Immunol. 2019 Mar 12;10:463. doi: 10.3389/fimmu.2019.00463 (PMC6423162; doi:10.3389/fimmu.2019.00463)
Supplement: Supplementary file 1 [file Data_Sheet_1.PDF]

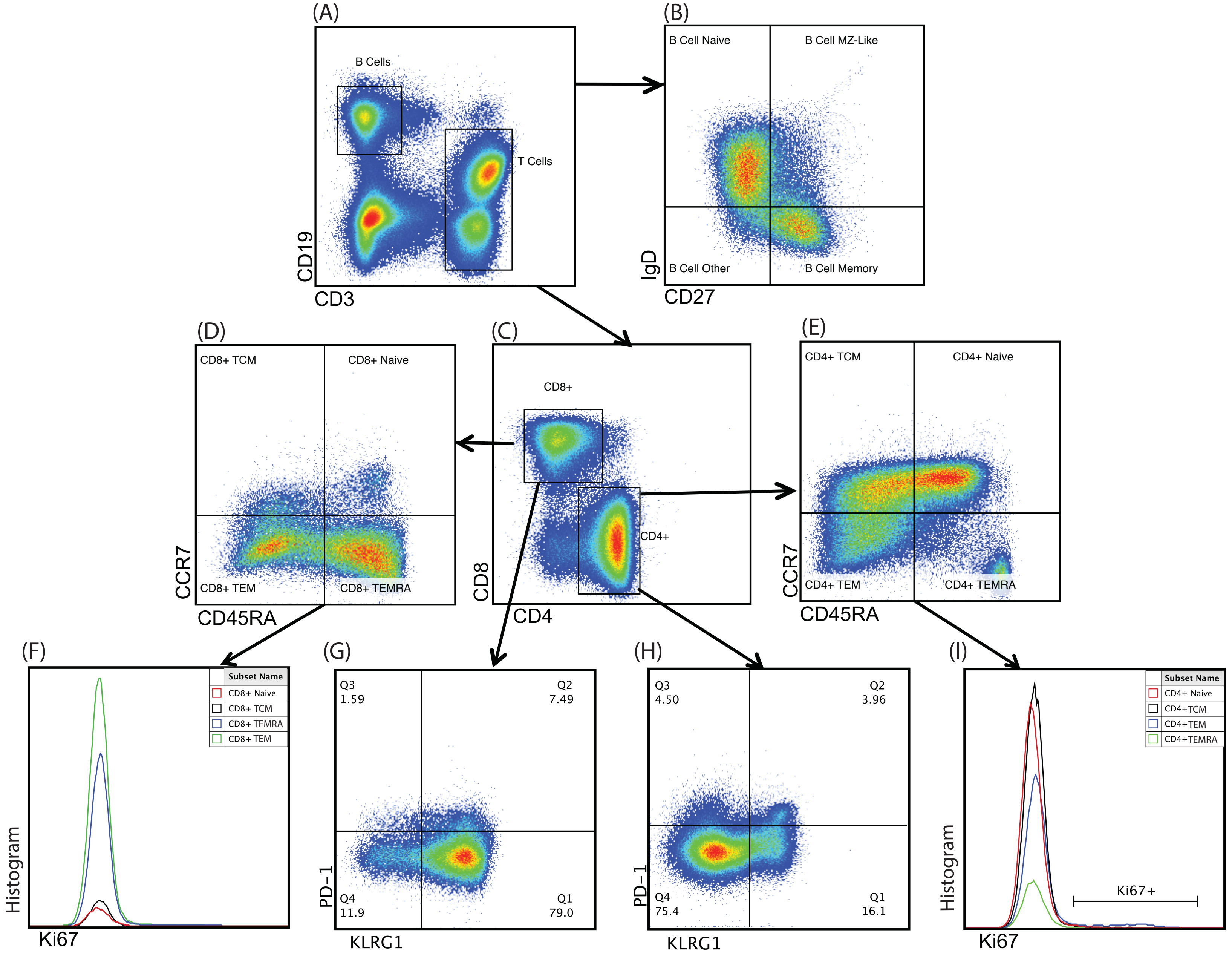

Supplemental Figure 1: Represntative gating strategy for identifying circulating T and B cells. (A) Total T cells were identified as (CD3+ CD19-) and to total B cells as (CD3- CD19+). (B) B cells were then further subdivided into; Naive B cells (CD27- IgD+), Memory B cells (CD27+ IgD-), MZ-like B cells (CD27+ IgD+). (C) T cells were first subdivided into Helper T cells (CD4+ CD8-), and Cytotoxic T cells (CD4- CD8+). (D,E) Helper and cytotoxic T cells were then diveded into Naive (CCR7+ CD45RA+; Naive), Central memory (CCR7+ CD45RA-; TCM), Effector memory (CCR7- CD45RA-; TEM), and Transitional effector memory (CCR7- CD45RA+; TEMRA). Proliferation of CD4+ (F) and CD8+ (I) T cell subsets was measured using Ki-67. Activation and exhaustion of CD4+ (G) and CD8+ (H) T cells was measured using KLRG1 and PD-1 respectively.

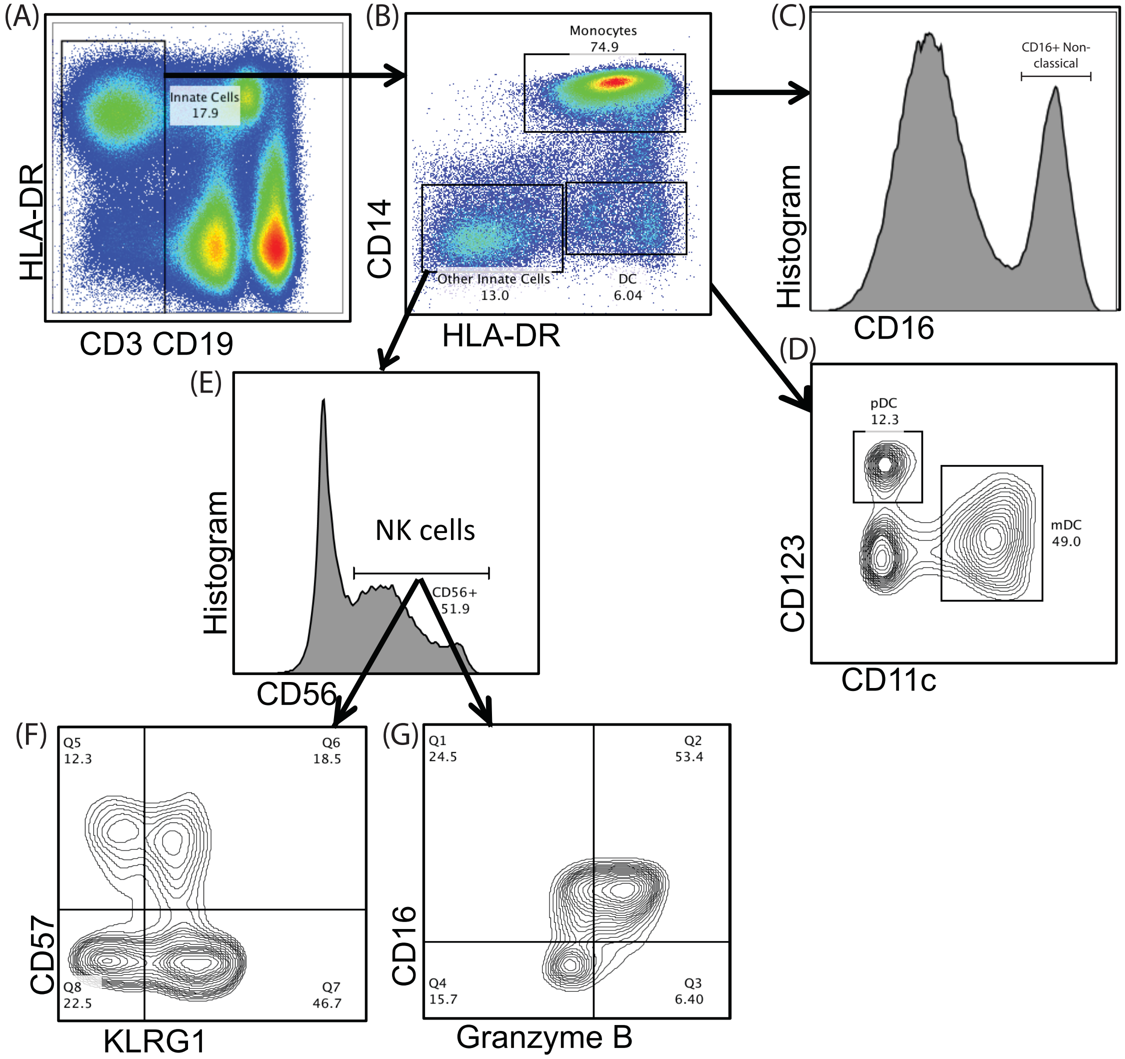

Supplemental Figure 2: Representative gating strategy for identifying circulating innate immune cells. (A) Innate immune cells were identfied by first gating out lymphocytes (CD3- CD19-). (B) Monocytes were identified as CD14+ HLA-DR+; Dendritic cells (DC) as CD14- HLA-DR+; and "other" innate cells as CD14- HLA-DR-. (C) Monocytes were further subdivided into Classical monocytes (CD14+ HLA-DR+ CD16-), and Non-classical monocytes (CD14+ HLA-DR+ CD16+), (D) DCs were further subdivided into Myleoid DC (CD14+ HLA-DR- CD123- CD11c+; mDC), and Plasmaytoid DC (CD14+ HLA-DR- CD123- CD11c+; pDC). (E) Natural Killer (NK) cells were idetified as CD14-HLA-DR-CD56+. NK cells we further subdivided based on the expression of activation markers KLRG1 and CD57 (F), as well as CD16 and Granzyme B+.

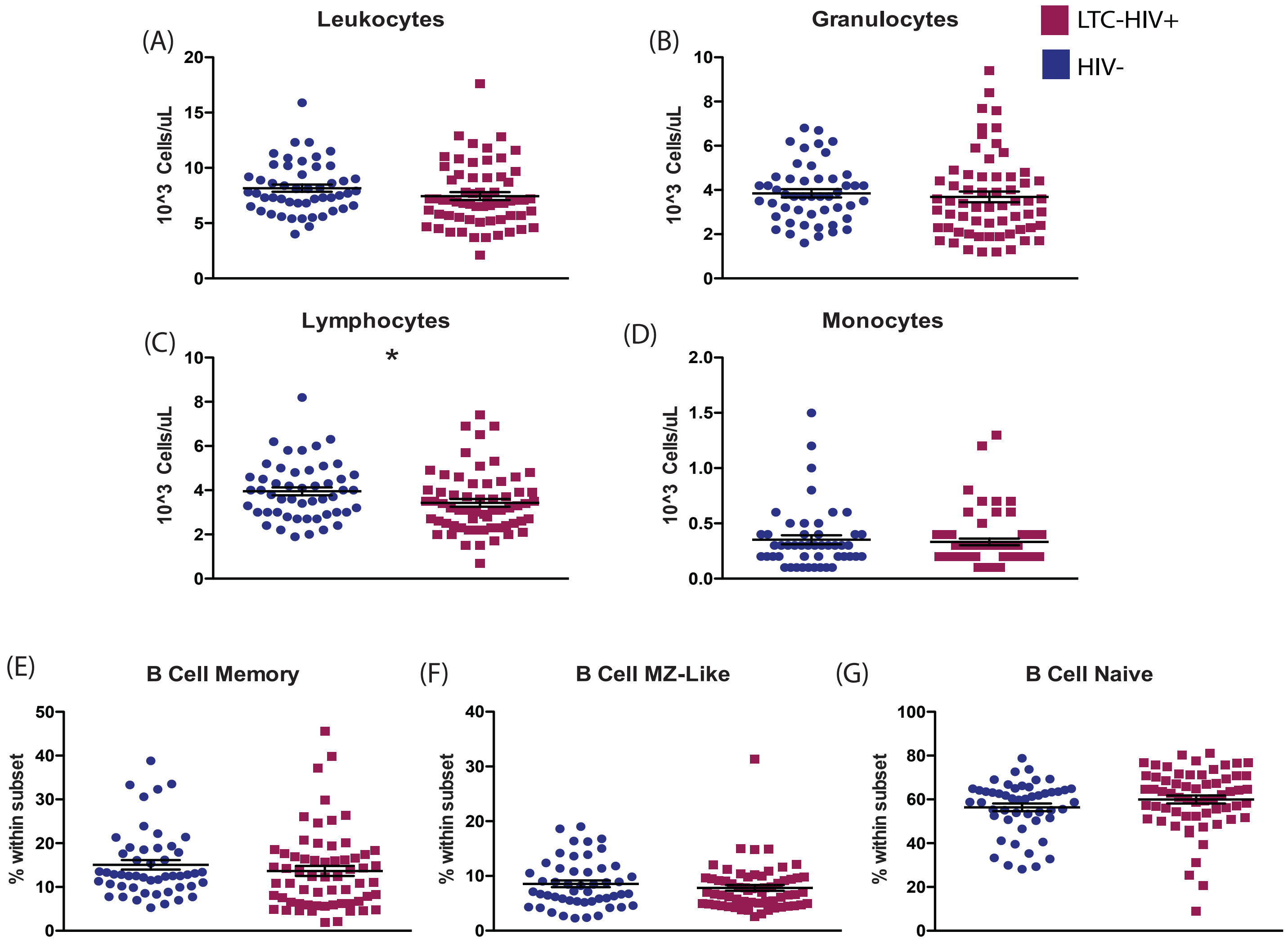

Supplemental Figure 3: Impact of LTC-HIV on circulating immune cells. complete blood cell counts of (A) total leukocytes (B) granulocytes (C) lymphocytes and (D) monocytes. Relative abundance of (E) memory B cells (F) MZ-like B cells and (G) Naïve B cells. Significance was determined using an unpaired t-test. * = $p < 0.05$.

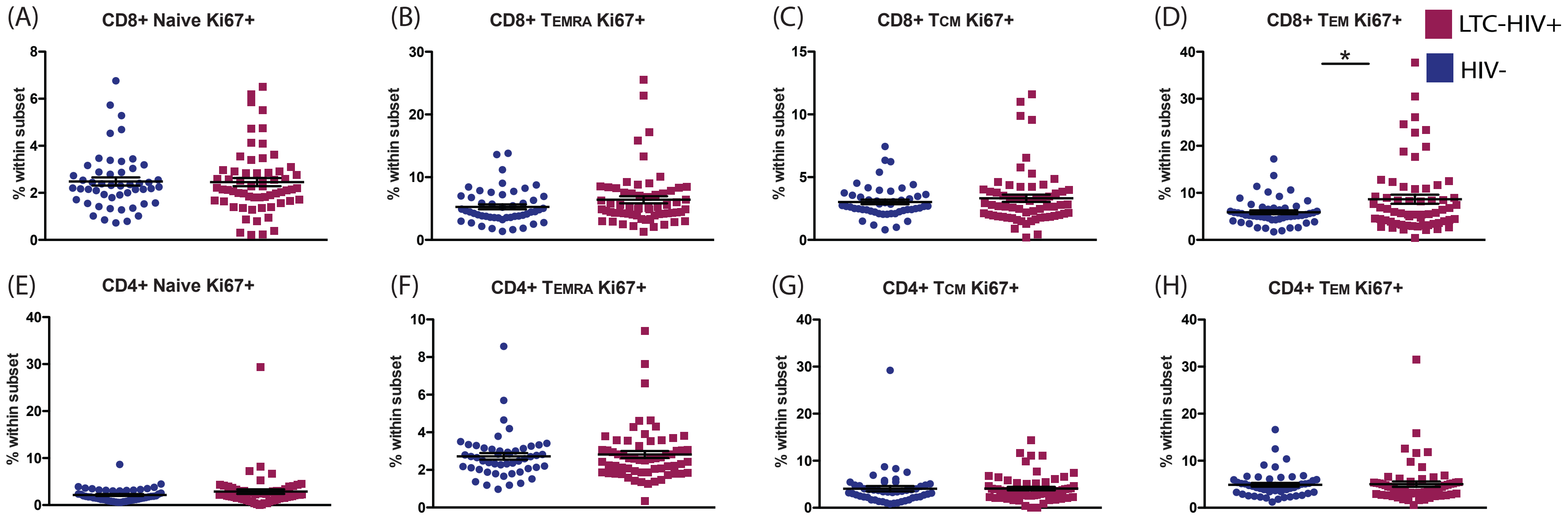

Supplemental Figure 4: Homeostatic proliferation of CD8+ and CD4+ T cell subsets. Percent of (A) Naive CD8+ T cells, (B) Transitional effector memory CD8+ T cells, (C) Central memory CD8+ T cells, (D) Effector memory CD8+ T cells, (E) Naive CD4+ T cells, (F) Transitional effector memory CD4+ T cells, (G) Central memory CD4+ T cells, and (H) Effector memory CD4+ T cells expressing Ki67. Significance was determined using an unpaired t-test. * = $p < 0.05$.

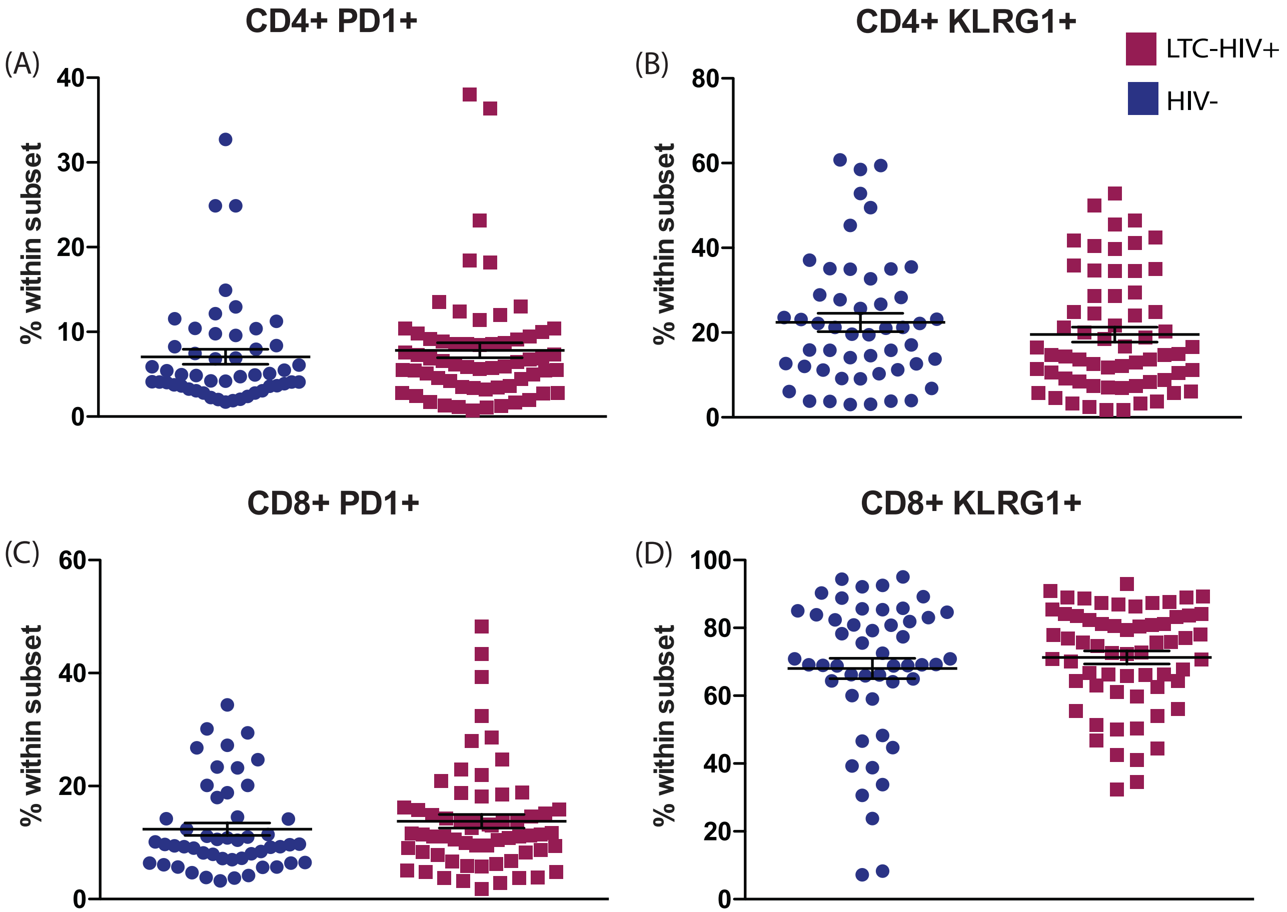

Supplemental Figure 5: Impact of LTC-HIV on T cell activation. Percent abundance of (A)PD1+ and (B) KLRG1+ cells within the CD4+ T cell subset; as well as (C) PD1+ and (D) KLRG1+ cells within the CD8+ T cell subset.

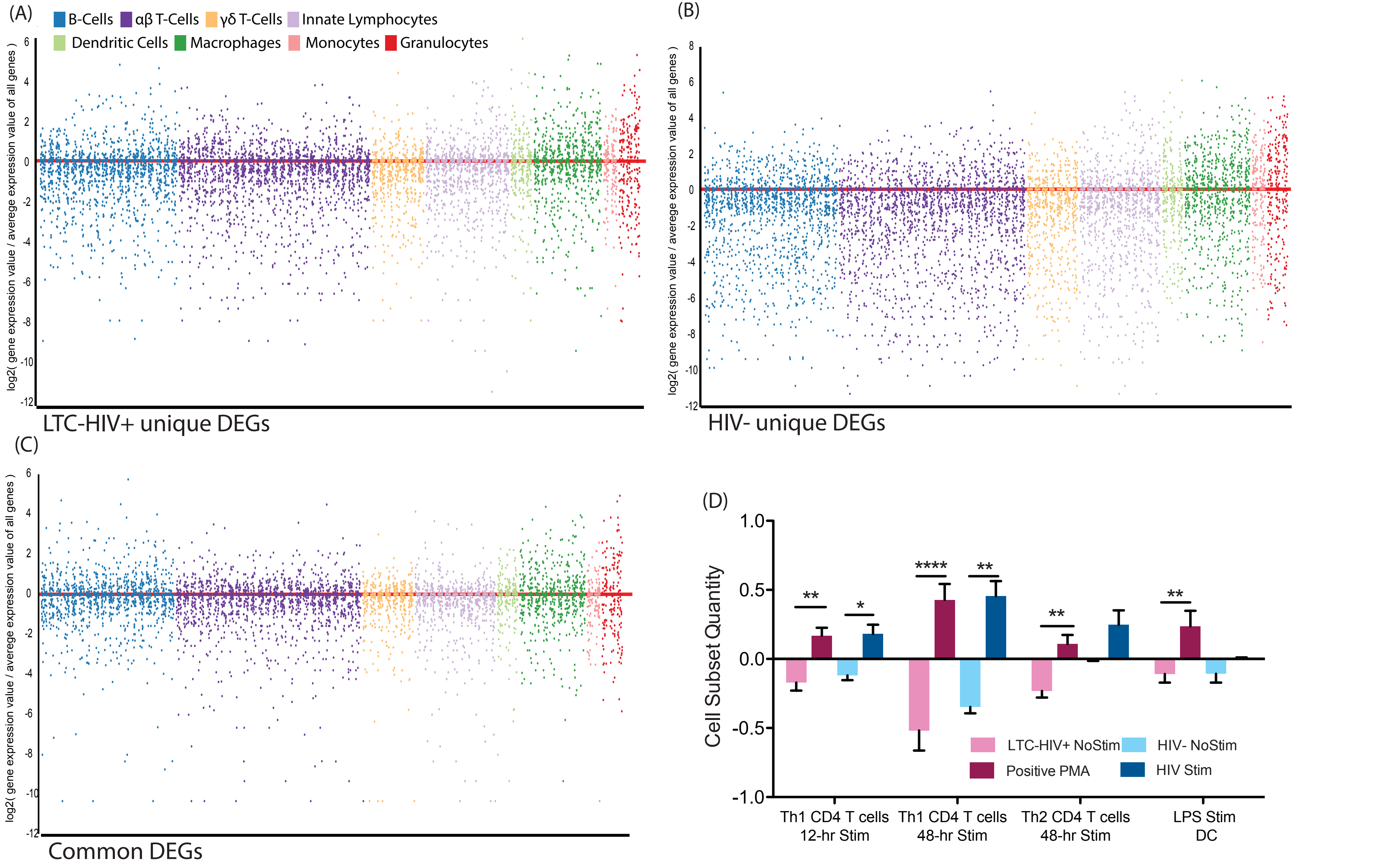

Supplemental Figure 6: ImmGen analysis of stimulation response DEGs and ImmQuant analysis of total transcriptional profiles. Heatmaps of showing expression profiles of DEGs detected following PMA stimulation in (A) HIV+ samples only (B) HIV- samples only and (C) Both HIV+ and HIV- samples across various immune cell populations as predicted by ImmGens' MyGeneSet application. Each dot represents a gene; genes with a positive relative expression value (above the "0" line) have a high likelihood of expression by the indicated immune cell subset while genes with negative relative gene expression value have low likelihood of expression by the indicated immune cell subset. (D) Immquant software was used to predict changes in immune cell populations upon stimulation. Significance was determined using a 1-way ANOVA with post-hoc Sidak multiple comparisons test with multiplicity adjusted P-values. * = $p < 0.05$, ** = $p < 0.01$, **** = $p < 0.0001$.

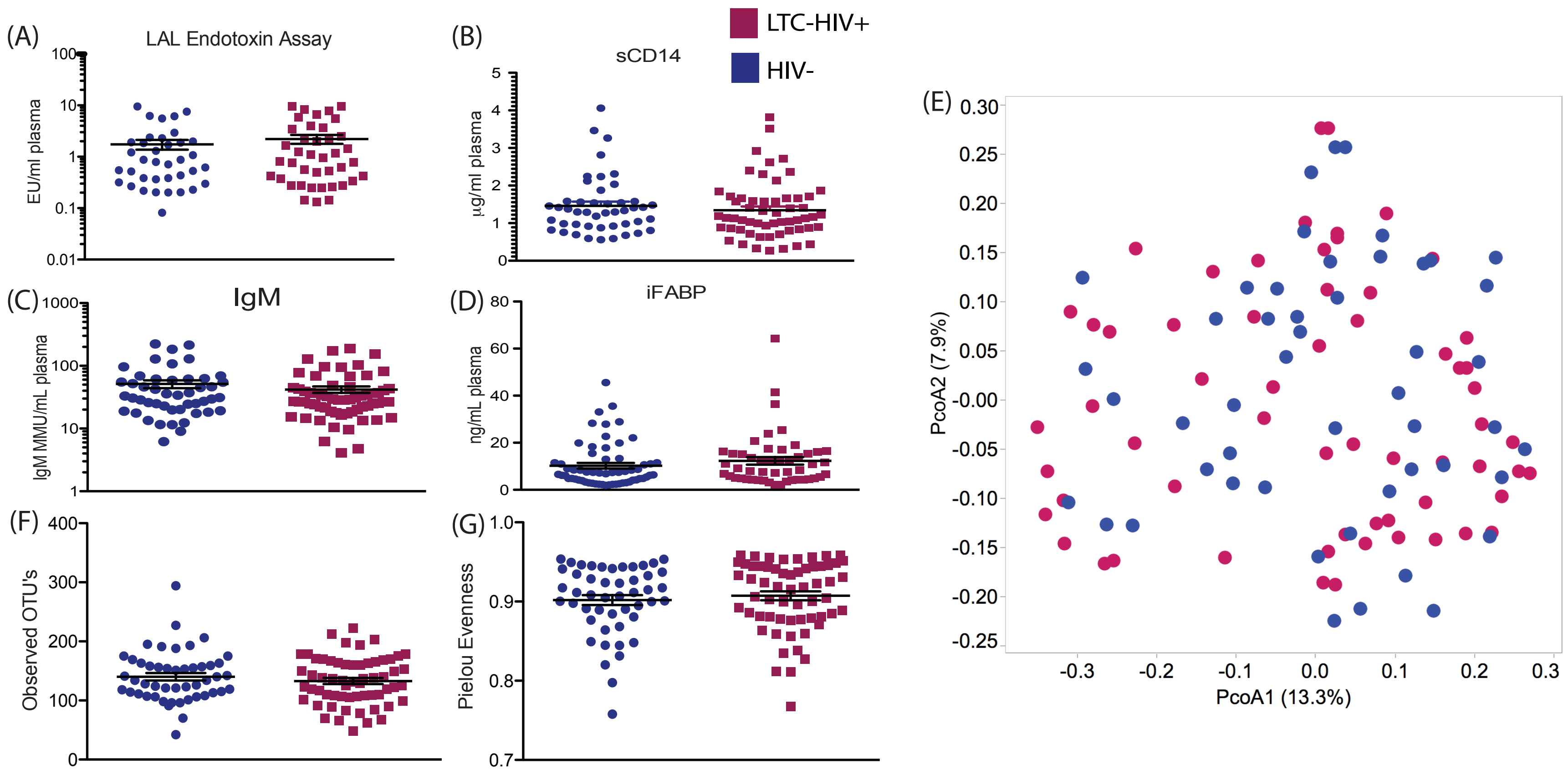

Supplemental Figure 7: No significant differences were observed in markers of microbial translocation or overall gut microbial community structure. (A) Limulus amebocyte lysate (B) soluble CD14 (C) IgM bound endotoxin (D) Fatty acid binding protein. (E) Principal coordinate analysis of unweighted UniFrac distance. Alpha-diversity measured by (F) observed operational taxonomic units (OTU's) and (G) pielou evenness.
